# The metal post material influences the performance of artefact reduction algorithms in CBCT images

**DOI:** 10.1590/0103-6440202204222

**Published:** 2022-03-07

**Authors:** Amanda Farias-Gomes, Rocharles Cavalcante Fontenele, Lucas P. Lopes Rosado, Frederico Sampaio Neves, Deborah Queiroz Freitas

**Affiliations:** 1 Department of Oral Diagnosis, Division of Oral Radiology, Piracicaba Dental School, University of Campinas, Piracicaba, SP, Brazil; 2 Department of Propedeutics and Integrated Clinic, Division of Oral Radiology, School of Dentistry, Federal University of Bahia, Salvador, BA, Brazil.

**Keywords:** Artefacts, cone-beam computed tomography, endodontics, metals

## Abstract

This study aimed to assess the effect of the MAR tool on the expression of artefacts in different regions of a tooth restored with different types of metal posts. Alveolar sockets (anterior, and posterior region) of a mandible and an unirradicular tooth were used. Cone beam computed tomography scans of the tooth without a metal post, and with cobalt-chromium (Co-Cr), nickel-chromium (Ni-Cr), or silver-palladium (Ag-Pd) were individually obtained, with 2 MAR conditions: disabled, and enabled. In an axial reconstruction, lines of interest (LOIs) were set around the canal: 4 in oblique (mesiobuccal, distobuccal, mesiolingual, distolingual) directions, and 4 in orthogonal (mesial, distal, buccal, lingual) directions. Beam-hardening artefacts expression was determined by calculating the difference in the mean of gray values (DMGV) between the experimental and control groups for each LOI. There was no significant difference in the DMGV values between “without MAR” and “with MAR” for any LOI, in neither anterior nor posterior mandible (p>0.05), for the Ni-Cr and Co-Cr groups. For the Ag-Pd, significant differences in the DMGV values were observed between “without MAR” and “with MAR” for most LOIs (p<0.05), mainly in oblique directions in the anterior region, and mesio-distal direction in the posterior region. MAR acted mostly in hypodense artefacts (negative DMGV). The effectiveness of the MAR tool of the OP300 CBCT unit varied according to the post material tested. It was effective in reducing the expression of artefacts raised by the Ag-Pd post, mainly in the tooth regions affected by hypodense artefacts, regardless of the mandibular region.

## Resumo

O objetivo nesse estudo foi avaliar o efeito da ferramenta de redução de artefato (FRA) na expressão de artefatos em diferentes regiões de um dente restaurado com diferentes tipos de pinos metálicos. Foram usados ​​alvéolos (regiões anterior e posterior) de uma mandíbula e um dente unirradicular. Imagens de tomografia computadorizada de feixe cônico do dente sem pino metálico intracanal e com pinos de cobalto-cromo (Co-Cr), níquel-cromo (Ni-Cr) ou prata-paládio (Ag-Pd) foram individualmente obtidas, com 2 condições de FRA: desativada e ativada. Em uma reconstrução axial, linhas de interesse (LOIs) foram definidas ao redor do canal: 4 em direções oblíquas (mésio-vestibular, disto-vestibular, mésio-lingual e disto-lingual) e 4 em direções ortogonais (mesial, distal, vestibular e lingual). A expressão dos artefatos de endurecimento do feixe foi determinada pelo cálculo da diferença na média dos valores de cinza (DMVC) entre os grupos experimental e controle para cada LOI. Não houve diferença significativa nos valores de DMVC entre as condições “sem FRA” e “com FRA” para nenhuma das LOIs, nem na região anterior nem na região posterior da mandíbula (p>0,05), para os grupos Ni-Cr e Co-Cr. Para o Ag-Pd, diferenças significativas nos valores de DMVC foram observadas entre “sem FRA” e “com FRA” para a maioria das LOIs (p<0,05), principalmente nas direções oblíquas na região anterior, e na direção mesio-distal na região posterior. A FRA atuou principalmente em artefatos hipodensos (DMVC negativos). A eficácia da FRA disponível no aparelho de TCFC OP300 variou de acordo com o material do pino testado. Ela foi efetiva na redução da expressão de artefatos originados por pinos de Ag-Pd, principalmente nas regiões do dente afetadas por artefatos hipodensos, independentemente da região mandibular.

## Introduction

Cone beam computed tomography (CBCT) has allowed great advances in diagnosis and planning in Dentistry due to its three-dimensional nature, with the representation of maxillofacial structures without image overlapping ^1^. Nevertheless, one of its greatest disadvantages is the formation of artefacts, which impair image quality and can hamper the recognition of several alterations and diseases. Amid the variety of artefacts in CBCT, those originating from the hardening of the X-ray beam, caused by materials of high atomic number and high-density, such as metal posts, may critically impact diagnoses of endodontic interest ^2^ ^,^ ^3^.

Prior researches have demonstrated a decrease in diagnostic values, specially of root fractures, when intracanal materials were present in the evaluated tooth ^2^ ^,^ ^4^ ^,^ ^5^. Such finding is probably related to the fact that the beam-hardening artefacts are depicted in the image as hyperdense bands, which may cover a fracture line, and as hypodense bands, which may simulate the presence of a root fracture ^3^ ^,^ ^6^ ^,^ ^7^. Moreover, it has been shown that the expression (hypodense or hyperdense) of the artefact in a tooth varies according to the intracanal material, the tooth position in the dental arch (anterior or posterior), and CBCT machine used ^8^.

Due to the negative impact of image artefacts, several studies have attempted to decrease or eliminate them by testing pre- (tube current, kilovoltage, number of basis images, voxel size, and field of view - FOV) and post-acquisition (metal artefact reduction - MAR - algorithms) variables ^3^ ^,^ ^8^ ^,^ ^9^ ^,^ ^10^ ^,^ ^11^. The MAR tool corresponds to algorithms developed by producers of some CBCT to enhance image quality throughout the reconstruction process, decreasing beam-hardening artefacts. It stands out in relation to the other variables, as it reduces the variance of gray values and elevates the contrast-to-noise ratio in the images without influencing the dose of radiation delivered to the patient ^10^ ^,^ ^12^.

Despite the effectiveness of MAR in improving image quality objectively, there is controversy regarding its efficacy on the diagnosis, since its use has demonstrated little or no effect on the diagnosis of various clinical conditions, e.g. periodontal defects, fractured endodontic tools, and root fractures ^7^ ^,^ ^13^ ^,^ ^14^ ^,^ ^15^ ^,^ ^16^. According to the consulted literature, the studies that objectively assessed the performance of the MAR tool have conducted such analyses on acrylic phantoms ^10^ ^,^ ^17^ ^,^ ^18^ or in regions not corresponding to the teeth ^12^ ^,^ ^19^. Therefore, it would be of interest to comprehend the effect of the MAR tool on different regions of the tooth to elucidate why these algorithms have demonstrated a positive effect on the objective image quality, but not on diagnosis. Additionally, it would be important to understand the influence of different metal post materials and regions of the dental arch on the MAR performance. Thus, the aim in this study was to assess the effect of the MAR tool on the expression of artefacts in different regions of a tooth restored with different types of metal posts, in CBCT images.

## Material and methods

This research was conducted after approval by the local research ethics committee (protocol CAAE 29004820.9.0000.5418).

The sample size was calculated in the Biostat software (version 5.3, Instituto de Desenvolvimento Sustentável Mamirauá, Tefé, MA, Brazil) and was based on the minimum difference among the groups, and the mean SD, adopting a statistical power of 0.75. Thus, the study sample was composed of three CBCT scans per group.

### Sample preparation

The research sample consisted of one single-rooted human tooth, and one edentulous dry human mandible. The tooth was cleaned with 70% alcohol, had calculi and soft tissue residue removed by ultrasonic scaling, and went through clinical and radiographic evaluation to verify the absence of root fractures, resorptive lesions, canal treatment, calcification, open apex, and dental anomalies. Then, with a diamond disc saw (Isomet 1000, Buehler Ltd., Lake Bluff, USA), the crown was sectioned at the cementoenamel junction.

Then, the root canal was instrumented to its entire length with a Mtwo NiTi rotatory system (VDW GmbH, Munich, Germany), using the protocol (size/taper) 10/0.04, 15/0.05, 20/0.06, and 25/0.06, and irrigation with distilled water ^8^. Posteriorly, the first two-thirds of the root were assessed with a number 2 piezo drill (Peeso Long Drill, Dentsply Sirona, Canada) for metal post placing. The metal posts - cobalt-chromium (Co-Cr), nickel-chromium (Ni-Cr), and silver-palladium (Ag-Pd) - were made by molding the root canal with a regular pin of Duralay acrylic resin. All metal posts had the same thickness and length (20 mm).

The dry mandible was used as a phantom for tooth accommodation. To do so, two alveolar sockets (right central incisor, and right first molar) were enlarged with a #1016 round diamond bur (KG Sorensen, Brazil) to fit the tooth passively. Previously to the CBCT scanning, the mandible was attached to the bottom of a plastic canister (16 cm diameter) filled with water. The water was employed to mimic the attenuation of X-rays by the soft tissues ^8^. At distinct times, the tooth was placed into the enlarged alveolar sockets to obtain the CBCT images.

### Image acquisitions

All scans were acquired with an OP300 Maxio (Instrumentarium Dental, Tuusula, Finland) device set at: 0.2 mm voxel size, 8 x 6 cm FOV, 90 kVp, 5 mA, 17.4 s scanning time, and 234 frames.

**Figure 1:**
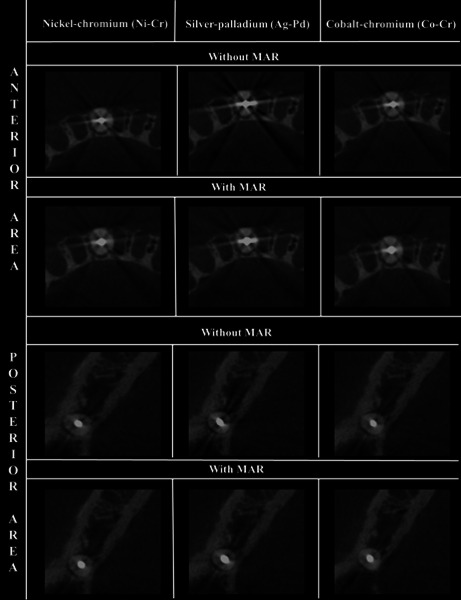
Cropped CBCT reconstructions (axial view) of each metal post (nickel-chromium, silver-palladium, and cobalt-chromium posts) tested, according to the MAR mode (with or without activation) and mandibular region.

The plastic canister containing the set “mandible + tooth” was fixed in the platform of the CBCT unit, and the position of the mandible was standardized by the reference lights of the machine. For each region of the mandible (anterior and posterior) and condition of MAR (enabled and disabled), four image sets were obtained according to the studied metal post. At first, scans of the tooth without the post placed inside the root canal (control group) were obtained. After, scans of the groups - Ni-Cr, Co-Cr, and Ag-Pd posts - were acquired (Figure 1). The posts were passively and individually placed inside the root canal, and all scans were obtained without stirring the phantom from its initial location in the machine. Each protocol was replicated 03 times for reproducibility, resulting in 48 CBCT images (4 tested groups x 2 mandibular regions x 2 conditions of MAR x 3 repetitions). During the image acquisitions, the phantom and the platform of the CBCT unit remained static to assure standardization of the position of the phantom during all scans. The MAR tool was applied for each scan, individually, right after obtaining the image with the “without MAR” protocol, without the need to acquire a new scan. The OP300 Maxio CBCT unit have two modes of MAR tool activation: one allows acquiring the scan with the MAR activated, and the other to acquire the scan without the MAR and then retrieve it with the MAR activated, with identical effects on the image ^19^.

### Image assessment

Images were exported as 8-bits TIFF files to the Image J software v.1.51 (National Institutes of Health, Bethesda, USA). They were individually assessed by an oral radiologist experienced in the objective assessment of image quality, in a calm and dark room.

**Figure 2.**
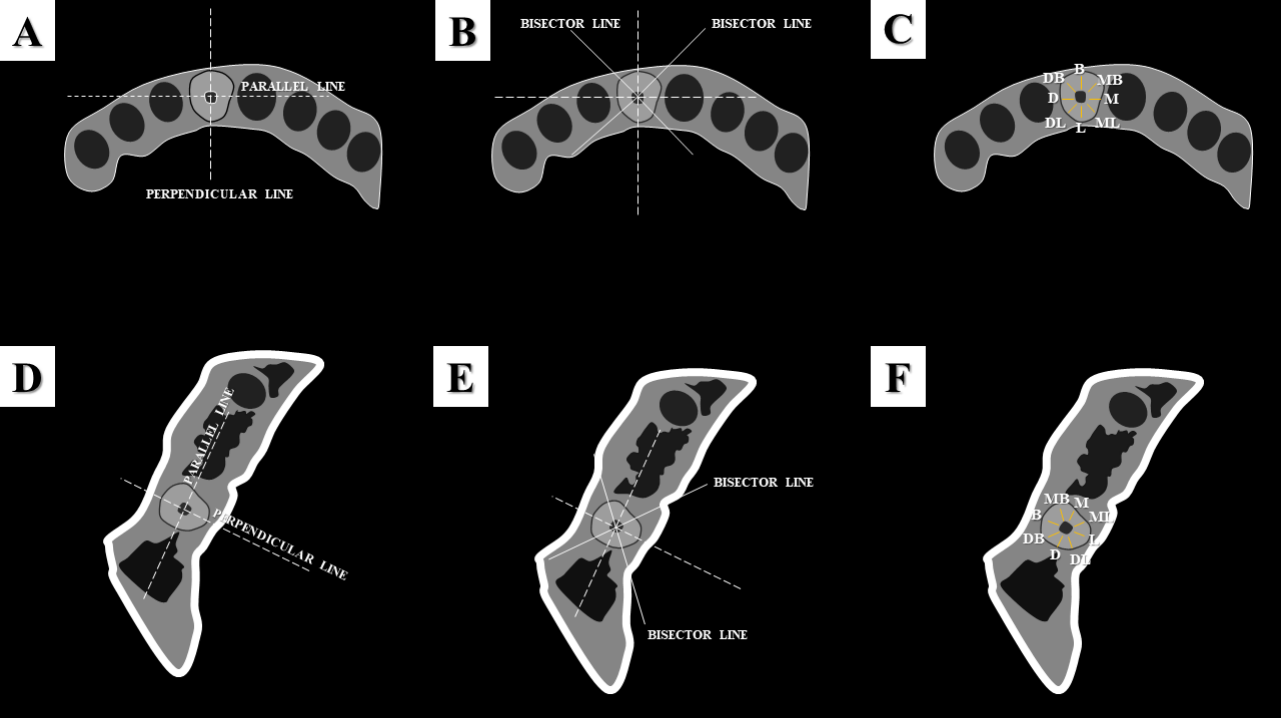
Schematic representation of the objective analysis according to the region of the mandible (anterior or posterior). (A-D) First, for each CBCT scan, the axial reconstruction at the middle level of the root was selected. Two lines were determined: one line crossing the center of the root canal, following the mandible’s long axis, and a line perpendicular to the first one. (B-E) At the intersection of the previously determined lines, two bisector lines (45º) were drawn. Eight LOIs (1.3mm-length, each) were set on the perpendicular, parallel and bisector lines: four LOIs were established in the orthogonal orientations (buccal, lingual, mesial and distal regions of the root), and four LOIs were established in the oblique directions (mesiobuccal, distobuccal, mesiolingual and distolingual regions of the root). LOIs, Lines of interest.

For analysis, the methodology established by Fontenele et al. was followed ^8^. Summarizing, the axial reconstruction at the middle level of the root was chosen for each CBCT scan. To do so, the first and last axial reconstructions in which the root was observed were used as guidance. On each middle level scan, 08 lines of interest (LOIs) (1.3mm-length, each) were demarcated around the root canal: 04 lines in oblique orientations (mesiolingual, distolingual, mesiobuccal, and distobuccal) and 04 lines in orthogonal directions (mesial, distal, buccal, and lingual), propagating from the outer surface of the post / root canal (control group) to the outer surface of the root (Figure 2 C and 2F).

To establish the LOIs, a line crossing the center of the root canal, and parallel to the mandible’s long axis, was drawn. Then, it was established a line perpendicular to the first one (Figure 2A-2D); at the encounter of these lines, 02 bisectorial lines (45°) were traced (Figure 2B-2E). The orthogonal LOIs were demarcated around the root canal, on the perpendicular and parallel lines, while the oblique LOIs were demarcated on the bisecting lines (Figure 2C and 2F). For every LOI, it was assessed the mean of the gray values. The beam-hardening artefacts expression was obtained by calculating the difference in the mean of gray values (DMGV) between the experimental groups (with metal post) and the control group (without metal post) for each LOI, within each post material. Thus, positive DMGV meant hyperdense artefacts, whereas negative DMGV meant hypodense artefacts. To standardize the establishment of the LOIs in the different scans, it was used the MACRO function of the ImageJ software. Also, the DMGV was acquired in a standardized manner, since both images, with and without MAR activation, were obtained without displacing the phantom from its initial spot in the CBCT unit. One-hundred and twenty days after the end of the image evaluation, the same evaluator assessed all CBTC scans and performed a new evaluation following the methodology previously described, to calculate the intra-examiner agreement.

### Statistical analysis

The intra-examiner agreement was calculated by the intraclass correlation coefficient (ICC) and interpreted according to the Cicchetti classification ^20^. Analysis of variance (*multi-way* ANOVA) with *post-hoc* Tukey’s test compared the DMGV to assess the effect of MAR also considering the other conditions that varied (post material and mandibular region) and their interactions. The null hypothesis assumed that the factors do not have an effect on the beam-hardening artefacts expression. Data were analyzed with the GraphPad Prism software v.7.0 (GraphPad Software, La Jolla, USA) and SPSS software v.24.0 (IBM Corp., Armonk, USA), with a significance level of 5 % (p-value < 0.05).

## Results

The ICC value was 0.99, showing an excellent intra-examiner agreement. Table 1 demonstrates the DMGV between the test (metal post) and control (without metal post) groups according to post material, artefact direction, mandibular region, and MAR condition.

For the Ni-Cr group, there was no significant difference between the “without MAR” and “with MAR” conditions for any direction, in neither the anterior nor posterior regions of the mandible (p>0.05), except for the mesial direction in the posterior region (p<0.05). A comparable trend was observed for the Co-Cr group, in which there were no significant differences between the “without MAR” and “with MAR” settings for most conditions (p>0.05), except for the buccal and distobuccal directions in the anterior region of the mandible (p<0.05). On the other hand, for the Ag-Pd post, significant differences were observed between “without MAR” and “with MAR” in most directions, in both the anterior (lingual, mesiobuccal, distobuccal, mesiolingual, distolingual) and posterior (mesial, distal, mesiobuccal, and distolingual) regions of the mandible (p<0.05). In general, when MAR was effective, there was a homogenization in the gray values, since for positive DMGV, there was a decrease in the mean of gray values, while for negative DMGV, there was an increase in the mean of gray values. It was observed that the MAR tool acted mostly in hypodense artefacts, except for two cases (lingual direction - anterior region of the mandible - in the Ag-Pd group, and in the buccal direction - anterior region of the mandible - in the Co-Cr group).

**Table 1:** Difference of the mean of gray values (DMGV) between the test (metal posts) and control (without metal post) groups according to the post material, mandibular region, artefact direction, and metal artefact reduction (MAR) condition.

	Direction	Mean of DMGV (Standard deviation)					
		Ni-Cr		Ag-Pd		Co-Cr	
		Without MAR	With MAR	Without MAR	With MAR	Without MAR	With MAR
Anterior region	Buccal	3.04 (3.65)	2.72 (3.53)	9.30 (2.63)	11.12 (1.94)	26.48 (30.10)	12.80 (0.12)*
	Lingual	15.51 (1.37)	13.22 (0.92)	17.23 (2.17)	4.55 (3.99)*	9.26 (1.17)	7.23 (1.11)
	Mesial	44.57 (5.46)	40.01 (4.99)	55.47 (4.44)	55.97 (1.25)	44.82 (0.50)	38.96 (1.91)
	Distal	64.88 (6.79)	59.66 (6.87)	74.85 (2.88)	73.89 (2.84)	70.05 (1.83)	65.16 (1.64)
	Mesiobuccal	-19.37 (5.11)	-15.44 (4.34)	-27.92 (2.06)	1.36 (3.40)*	11.88 (1.90)	13.82 (1.97)
	Distobuccal	-29.23 (2.96)	-17.59 (1.23)	-32.43 (3.87)	-0.49 (2.30)*	-23.26 (0.26)	-8.15 (2.05)*
	Mesiolingual	-33.36 (0.43)	-26.76 (2.59)	-40.64 (2.55)	-24.00 (3.36)*	-30.49 (1.49)	-24.14 (2.91)
	Distolingual	-11.83 (4.12)	-1.00 (2.75)	-40.67 (2.62)	-13.34 (6.22)*	-15.41 (1.68)	-12.61 (1.91)
Posterior region	Buccal	13.94 (2.42)	18.39 (2.58)	32.00 (1.98)	36.80 (1.18)	20.78 (2.72)	25.45 (2.46)
	Lingual	26.48 (1.01)	26.30 (1.03)	51.26 (2.58)	46.94 (1.60)	26.08 (2.32)	25.78 (1.36)
	Mesial	-16.54 (2.25)	-7.32 (2.08)*	-28.54 (3.46)	-17.15 (3.67)*	-18.67 (1.31)	-12.04 (1.25)
	Distal	-4.05 (1.92)	-0.35 (1.56)	-18.35 (2.98)	-4.73 (2.91)*	-3.07 (1.59)	2.19 (0.78)
	Mesiobuccal	-5.19 (5.77)	-0.89 (4.29)	-18.90 (5.66)	-10.37 (3.05)*	-8.87 (4.08)	-5.99 (3.69)
	Distobuccal	23.12 (2.80)	22.37 (2.21)	32.79 (2.30)	31.96 (2.65)	18.67 (1.02)	18.32 (0.75)
	Mesiolingual	38.27 (1.94)	37.53 (1.57)	43.62 (4.45)	40.54 (4.37)	30.72 (2.92)	31.80 (2.35)
	Distolingual	-6.07 (3.05)	-1.40 (3.18)	-7.55 (3.29)	3.52 (4.38)*	-1.57 (2.59)	2.76 (2.41)

* Significantly differed from “without MAR” within the same post material, direction, and mandibular region evaluated (p<0.05).

## Discussion

In the present study, it was aimed to assess the effect of the MAR tool on the expression of artefacts in different regions of a tooth restored with different types of metal posts, in CBCT images. It was found that the MAR tool performed differently depending on the metal post composition. In general, when the tooth was restored with a Ni-Cr or Co-Cr post, MAR was not effective, since there was no significant decrease in the artefact expression in the different regions of the tooth. However, the MAR tool acted positively in regions where artefacts (hyperdense or hypodense) were more pronounced, i.e., when the tooth was restored with an Ag-Pd post. Therefore, these findings can shed a light on the reason why previous studies ^21^ ^,^ ^22^ ^,^ ^23^ ^,^ ^24^ ^,^ ^25^ have reported that the MAR tool is not efficient in improving the diagnosis of VRF in teeth restored with metal posts.

The beam hardening phenomenon has been extensively studied in the literature. Its effects hamper the diagnosis of different conditions in Dentistry, mainly in Endodontics and Oral Rehabilitation. In addition to impacting the diagnosis of root fractures, as previously mentioned, the beam hardening phenomenon also influences measurements around dental implants by overestimating the implant diameter ^26^, making it difficult to detect gaps at prosthetic crowns ^27^; it also decreases the detection of internal root resorptions when adjacent teeth are restored with metal posts ^28^, impairs the diagnosis of misfits at the implant-abutment joint ^29^, and hamper the diagnosis of fractured endodontic instruments in teeth filled with gutta-percha ^30^.

Previous researches have also objectively investigated the expression of artefacts in teeth with metal posts ^8^ ^,^ ^31^ ^,^ ^32^; however, the methodologies used were different from that of the present study. Lira de Farias Freitas et al. ^32^ quantitatively evaluated the artefacts produced by two types of metal posts (Ni-Cr and Ag-Pd) by using the threshold of gray values that would best represent each type of artefact (hypodense or hyperdense). Although it is an effective analysis for the total quantification of the artefacts produced, this methodology does not allow to determine which regions of the tooth would be most affected by each type of artefact. Conversely, Fontenele et al. ^8^ proposed the mapping (i.e., analysis of the expression of the artefacts in different regions of the tooth) of artefacts generated by different metal posts (Ni-Cr, Ag-Pd, and Co-Cr). They used the mean of gray values to determine which areas of the tooth were more affected by artefacts, according to their intensity (hyperdense or hypodense). The intensity of the artefacts was obtained from the comparison between a control group (without metal post) and the experimental groups (with metal posts); however, the interpretation of the generated data is not direct, which can make it difficult for clinicians to understand their results. Differently, the objective analysis employed in the present study is novel and easy for clinicians to comprehend, as it is possible to know which regions of the tooth had hypodense (negative DMGV), or hyperdense artefacts (positive DMGV), from directly reading the results.

The objective assessment of the expression of artefacts generated by high-density materials (e.g. dental implants, metal posts, amalgam, and gutta-percha) has been broadly performed in the literature ^8^ ^,^ ^10^ ^,^ ^11^ ^,^ ^12^ ^,^ ^17^ ^,^ ^19^. In agreement with the present investigation, these previous studies have also made use of a single evaluator, since automated tools (e.g. MACRO and ROI manager of the ImageJ software) were used to perform the analyses. In our study, in addition to the use of the MACRO tool, the phantom and the platform of the CBCT unit remained immobile during all image acquisitions, assuring the standardization of the image analyses. Such standardization was demonstrated by the excellent values of reproducibility obtained.

The performance of the MAR algorithms has been investigated, both objectively and subjectively ^8^ ^,^ ^12^ ^,^ ^19^ ^,^ ^25^. It is a consensus that the MAR tool promotes some homogenization in the gray values in the areas most affected by the beam-hardening artefacts in an image ^11^ ^,^ ^12^ ^,^ ^19^. However, the MAR tool has demonstrated no effect, or even a negative influence, on several diagnostic tasks (e.g., VRF in teeth restored with metal posts) ^13^ ^,^ ^14^ ^,^ ^21^ ^,^ ^22^ ^,^ ^23^ ^,^ ^24^ ^,^ ^25^, which generates controversy between the objective and subjective studies on the effect of MAR on CBCT image quality, especially with regard to the diagnosis of VRF. As far as we know, there are no studies about the influence of the MAR tool on the artefact production in different regions of a tooth restored with distinct metal posts. Our results may explain why the MAR activation does not improve the accuracy in the diagnosis of VRF in teeth with metal posts, as we found that the MAR tool is ineffective in reducing the artefacts generated by Ni-Cr and Co-Cr alloys. These artefact-generator materials were commonly used in experimental studies that have evaluated the influence of different acquisition parameters (e.g., MAR) on the diagnosis of VRF ^3^ ^,^ ^5^ ^,^ ^14^. Thus, in face of our results, it may be expected that there will be no enhancement in the VRF diagnosis in teeth restored with Ni-Cr and Co-Cr alloys when MAR is enabled.

Therefore, regarding the effect of the different post materials and the MAR performance, an interesting result was observed in our research. The MAR tool was effective in promoting some homogenization in the gray values (i.e., reducing the positive DMGV values, and increasing the negative DMGV values) in several regions of the tooth restored with an Ag-Pd post, regardless of the mandibular region (anterior or posterior) tested. In contrast, little or no effect of MAR was observed in the Ni-Cr and Co-Cr groups. This different behaviour of the MAR algorithms in relation to the alloys may be explained by their distinct atomic numbers and physical densities. The Ag-Pd post has higher atomic number (ZAg=47 and ZPd=46) than the other alloys tested (ZNi=28; ZCr=23; ZCo=24; ZCr=27); likewise, according to the manufacturer’s information, the physical density of the Ag-Pd is higher (d=10.4 g/cm³) than that of the Ni-Cr (d=8.2 g/cm³) and Co-Cr (d=8.6 g/cm³) alloys, which leads to greater production of artefacts, due to the greater absorption of low energy X-rays, by the former material ^8^ ^,^ ^11^ ^,^ ^32^. In line with previous studies ^12^ ^,^ ^19^, we proved that the MAR tool performs better when there is greater production of artefacts, as observed in several regions of the tooth restored with Ag-Pd. Thus, although it was not our aim to directly compare the expression of artefacts among the post materials, the results of the present study may indicate that the choice of the metal post should not be based solely on the physicochemical properties of the material, but also on its potential to generate image artefacts and/or be positively impacted by the use of MAR. Nevertheless, further studies investigating the expression of artefacts and performance of MAR on diagnostic tasks involving teeth restored with different post materials are encouraged.

Another outcome of the present study is related to the type of artefact that was most affected by the MAR tool. Of all regions (n=12) influenced by MAR activation, only two had hyperdense artefacts (positive DMGV). The other regions (n=10) had hypodense artefacts (negative DMGV). Extrapolating for a clinical scenario, our results suggest that MAR would be more effective in decreasing the number of false-positive cases of VRF, as it was better in decreasing artefacts that mimic fracture lines. Conversely, MAR would not be so effective in reducing false-negative cases of VRF, as it was less effective in reducing hyperdense artefacts, which may cover fracture lines.

We recognize that the main guidelines on the applications of CBCT in endodontics ^33^ ^,^ ^34^ recommend the use of the smallest FOV and voxel size (high-resolution) available. However, in this study, we used a medium FOV (8 x 6 cm), as we needed to encompass the anterior and posterior aspects of the mandible in the same volume, even though different exams have been acquired to evaluate each region. As suggested by previous studies ^7^ ^,^ ^8^ ^,^ ^35^, the use of a medium FOV in this type of experimental model is recommended in order to standardize the structures inside and outside the FOV, thereby, avoiding negative and varied influence of the exomass in the evaluation of the gray values in structures located inside the FOV ^36^. About the voxel size, we selected the smallest voxel available for the selected acquisition protocol.

The present study had an in vitro design, which has inherent limitations. However, this was the only possible method to evaluate the various factors tested, as it is ethically and biologically unacceptable to expose a patient to radiation several times only for research proposes. However, to simulate a clinical scenario as accurately as possible, the images used in the present study were acquired using a phantom composed of a human mandible and a human tooth included in a container filled with water (i.e., simulating the attenuation of the X-rays by human soft tissues). A limitation of this study is that only the MAR algorithm available in the OP300 CBCT unit was tested. This equipment was chosen as it allows to obtain a scan without the application of MAR, and another one with the MAR applied, in the same acquisition, guaranteeing that the phantom was not moved during the acquisitions, which is essential for a trustworthy objective analysis. We suggest that future studies investigate the influence of other MAR algorithms on the expression of artefacts in teeth restored with metal posts.

In conclusion, the effectiveness of the MAR tool available in the OP300 CBCT unit varied according to the post material and artefact expression. It was effective in reducing the expression of artefacts generated by the Ag-Pd post, mainly in the areas affected by hypodense artefacts, regardless of the mandibular region. The same performance was not observed for the Co-Cr and Ni-Cr posts.
